# Lifelines NEXT: a prospective birth cohort adding the next generation to the three-generation Lifelines cohort study

**DOI:** 10.1007/s10654-020-00614-7

**Published:** 2020-02-25

**Authors:** Willemijn D. B. Warmink-Perdijk, Lilian L. Peters, Ettje F. Tigchelaar, Jackie A. M. Dekens, Soesma A. Jankipersadsing, Alexandra Zhernakova, Willem J. R. Bossers, Jan Sikkema, Ank de Jonge, Sijmen A. Reijneveld, Henkjan J. Verkade, Gerard H. Koppelman, Cisca Wijmenga, Folkert Kuipers, Sicco A. Scherjon

**Affiliations:** 1 Department of Midwifery Science, Amsterdam Public Health Research Institute, Amsterdam UMC, Vrije Universiteit Amsterdam, Van de Boechorstraat 7, 1081 BT Amsterdam, The Netherlands; 2 Department of General Practice and Elderly Medicine, University Medical Center Groningen, University of Groningen, Antonius Deusinglaan 1, 9713 AV Groningen, The Netherlands; 3 AVAG (Academy Midwifery Amsterdam and Groningen), Dirk Huizingastraat 3-5, 9713 GL Groningen, The Netherlands; 4 Department of Genetics, University Medical Center Groningen, University of Groningen, Antonius Deusinglaan 1, 9713 AV Groningen, The Netherlands; 5 Center for Development and Innovation, University Medical Center Groningen, University of Groningen, Antonius Deusinglaan 1, 9713 AV Groningen, The Netherlands; 6 Lifelines Cohort Study, Bloemsingel 1, 9713 BZ Groningen, The Netherlands; 7 Department of Health Sciences, University Medical Center Groningen, University of Groningen, Antonius Deusinglaan 1, 9713 AV Groningen, The Netherlands; 8 Department of Pediatrics, Pediatric Gastroenterology – Hepatology, University Medical Center Groningen, University of Groningen, Antonius Deusinglaan 1, 9713 AV Groningen, The Netherlands; 9 Department of Pediatric Pulmonology and Pediatric Allergy, Beatrix Children’s Hospital, University Medical Center Groningen, University of Groningen, Antonius Deusinglaan 1, 9713 AV Groningen, The Netherlands; 10 Groningen Research Institute for Asthma and COPD (GRIAC), University Medical Center Groningen, University of Groningen, Antonius Deusinglaan 1, 9713 AV Groningen, The Netherlands; 11 Department of Pediatrics/Laboratory Medicine, University Medical Center Groningen, University of Groningen, Antonius Deusinglaan 1, 9713 AV Groningen, The Netherlands; 12 Department of Obstetrics and Gynecology, University Medical Center Groningen, University of Groningen, Antonius Deusinglaan 1, 9713 AV Groningen, The Netherlands

**Keywords:** Biobank, Birth cohort, Prospective study, Microbiome, Transgenerational effects, Developmental Origins of Health and Disease (DOHaD)

## Abstract

Epidemiological research has shown there to be a strong relationship between preconceptional, prenatal, birth and early-life factors and lifelong health. The Lifelines NEXT is a birth cohort designed to study the effects of intrinsic and extrinsic determinants on health and disease in a four-generation design. It is embedded within the Lifelines cohort study, a prospective three-generation population-based cohort study recording the health and health-related aspects of 167,729 individuals living in Northern Netherlands. In Lifelines NEXT we aim to include 1500 pregnant Lifelines participants and intensively follow them, their partners and their children until at least 1 year after birth. Longer-term follow-up of physical and psychological health will then be embedded following Lifelines procedures. During the Lifelines NEXT study period biomaterials—including maternal and neonatal (cord) blood, placental tissue, feces, breast milk, nasal swabs and urine—will be collected from the mother and child at 10 time points. We will also collect data on medical, social, lifestyle and environmental factors via questionnaires at 14 different time points and continuous data via connected devices. The extensive collection of different (bio)materials from mother and child during pregnancy and afterwards will provide the means to relate environmental factors including maternal and neonatal microbiome composition) to (epi)genetics, health and developmental outcomes. The nesting of the study within Lifelines enables us to include preconceptional transgenerational data and can be used to identify other extended families within the cohort.

## Introduction

Exposures or events in the preconceptional, prenatal, birth and early life period may have lifelong effects on an individual’s development and disease susceptibility. An impressive body of evidence in the field of Developmental Origins of Health and Disease (DOHaD) has shown that maternal factors (e.g. physical and psychological health, lifestyle) and environmental factors can modulate the developmental program [1–3]. This modulation could then permanently change the physiology, metabolism, epigenome and microbiome of the child, subsequently affecting healthy development or increasing susceptibility to (chronic) diseases [4–6]. Developmental programming via epigenetic changes may also have transgenerational effects without changing the genetic code in either the maternal or paternal line [7].

Several birth cohorts have been initiated with comprehensive data collection that include questionnaires as well as biomaterials to identify factors associated with child health and disease susceptibility [8]. However, although the importance of both immune status and the microbiome for the development of diseases has been established in multiple human cross-sectional studies and in animal models, only a few cohort studies include comprehensive immunological and microbiome data [9]. Moreover, very few birth cohorts have included three or more generations in their study design.

Another unique feature of the Lifelines NEXT data set is the presence of biomaterials from homebirths. In the Netherlands, 13% of children of mothers with low risk of complication are born at home [10]. This provides a unique opportunity to study the influence of homebirth versus birth in the hospital environment (with or without medical interventions) on the development of the newborn child. Previous work has found associations of birth interventions with increased risks of several immune-related diseases [11], but the mechanism(s) underlying this observation remain unclear. Data collected in Lifelines NEXT could assist in uncovering these mechanisms.

Finally, compared to other cohorts, Lifelines NEXT will collect large volumes of breastmilk over a long period of time [8]. This provides the means to study the mechanisms behind the immune-competent proteins in breastmilk, the large amounts of cholesterol it contains and the different molecular structures of its lipid content compared to infant formulas. Cellular trafficking from mother to child (“chimerism”) and (favorable) antigen exposition in an immune-modulated neonatal environment have been suggested to be part of mechanisms that have a long-lasting effect on neonatal health and can be studied in Lifelines NEXT [12–15].

Lifelines NEXT is an observational prospective birth cohort with a transgenerational design of up to four generations. Lifelines NEXT is embedded within the previously described Lifelines cohort study [16, 17]. In short, Lifelines is a prospective population-based cohort study comprising approximately 10% of the population of the Northern Netherlands (the provinces of Groningen, Drenthe and Friesland). Lifelines includes 167,729 participants and was designed as a three-generation cohort [16, 17]. Within Lifelines, 112,596 participants (67%) have a known family member within the cohort, with 84,888 (51%) part of two generations and 20,362 participants (12%) part of three generations of Lifelines participants [16]. Moreover, once genetic information becomes available, we anticipate that more extended families will be identified within the cohort given the expected cryptic genetic-relatedness of the more homogenous northern part of the Netherlands [18]. Initially, only children 8 years and older participated in Lifelines, and no data and materials were collected from Lifelines participants who became pregnant. With the initiation of Lifelines NEXT, we are filling this gap and adding a fourth generation to Lifelines.

The primary study objective of Lifelines NEXT is to investigate the effects of early life or pre-conceptional transgenerational events on health in early childhood. Its secondary aim is to correlate genomic, epigenetic, serological, metabolomic, microbiome, medical, social and environmental factors to early life health. Lifelines NEXT will provide unique opportunities to separate non-genetic from genetic familial transmission and to assess (epi)genetic influences and imprinting. Moreover, Lifelines NEXT can associate exposures in the preconception, prenatal, birth and early life period with healthy development and (chronic) disease susceptibility. The main risk factors of interest include microbiome, (epi)genetic, environmental and lifestyle factors.

This paper describes the infrastructure of the detailed and unique (bio)data collection of Lifelines NEXT, which starts in early pregnancy (as early as 12 weeks gestational age) and follows the offspring extensively up until at least 12 months of age. The infrastructure of Lifelines will be used to extend children’s follow up and offers participants the opportunity to enter the regular Lifelines cohort including its standardized data collection of biomaterials and questionnaires [16, 17].

## Methods

### Participants and recruitment strategy

Lifelines NEXT aims to include 1500 pregnant women. Upon inclusion, their partners are also invited to participate. Children are included in the birth cohort on the day they are born.

**Fig. 1:**
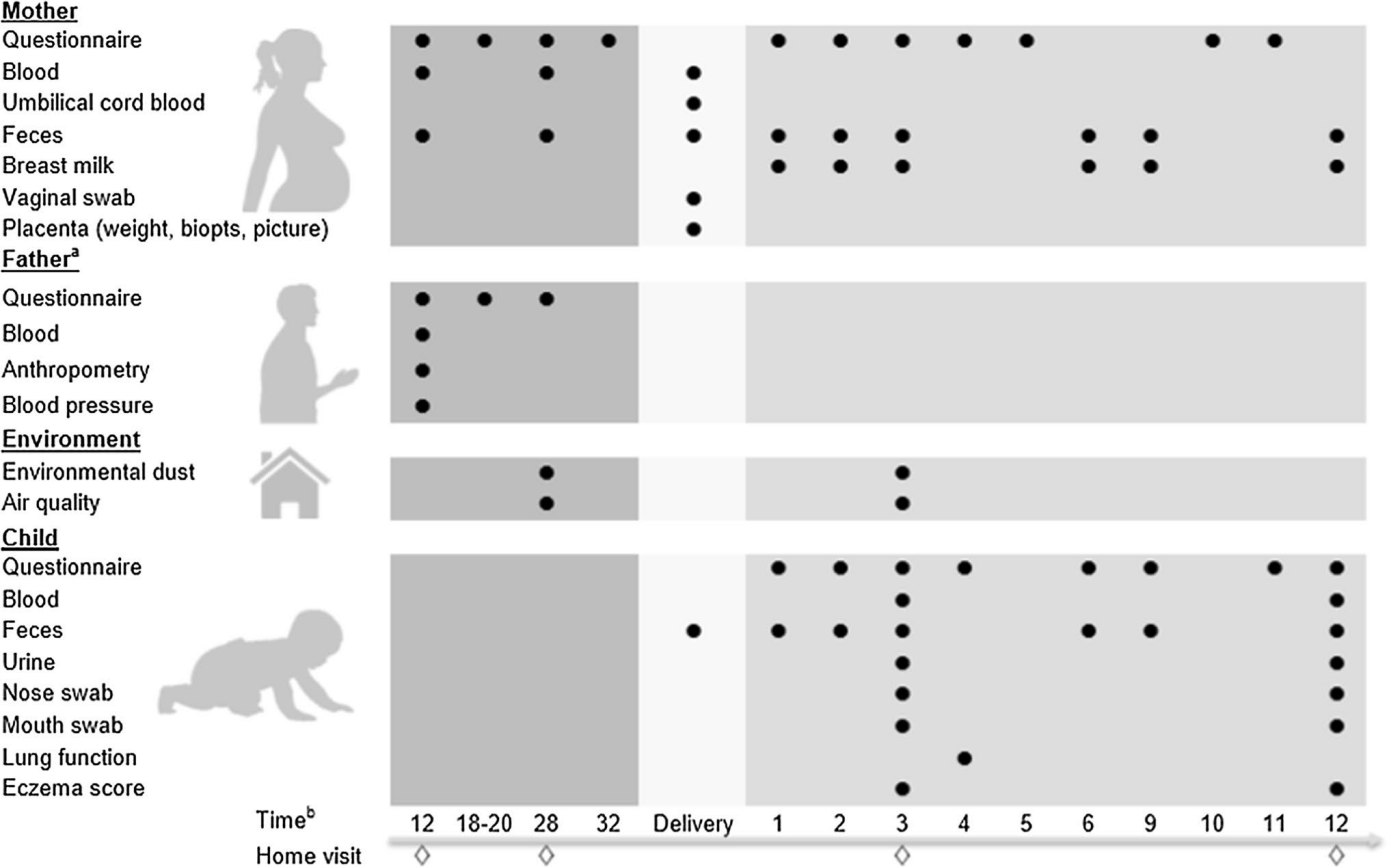
Timeline of the Lifelines NEXT study indicating the data collection per time point. ^a^Only for non-Lifelines fathers. Measurements start at the moment of inclusion. ^b^Gestational age (weeks)/child’s age (months)

From 2016 to 2021, Lifelines NEXT will recruit pregnant women in the Northern part of the Netherlands, preferably from 12 weeks gestational age onwards. Eligible women are recruited via midwives or gynecologists, the Lifelines website, Lifelines NEXT social media (announcements on Facebook and Instagram), Lifelines newsletters, pregnancy-related events and informational meetings. A leaflet containing details about Lifelines NEXT is provided. Pregnant women who consent to participate in the study are contacted via the Lifelines service desk. A research assistant at the service desk then provides more detailed information about the data collection of Lifelines NEXT (Fig. 1) and explains the informed consent procedure. A research nurse is allocated as the primary contact person for each Lifelines NEXT participant and obtains the informed consent at the first home visit. Both parents need to consent that their child will be included in Lifelines NEXT.

### Procedures for gathering and storage of (bio)materials and data

Questionnaire data and extensive biomaterial data will be collected for the mother, father, child and the family home environment (Fig. 1). Data collection starts, preferably, at 12 weeks of gestation. Research nurses will visit participants four times to perform tests and collect (bio)materials. To minimize the burden on the participants, sample collection is performed at the participant’s home by the research nurse, by the mother herself or by her maternity care provider. This participant-based sampling program was designed using insights and experience gathered in similar successful program like Lifelines DEEP [19]. To assist participants in this process, Lifelines NEXT provides standardized protocols for the collection and storage of (bio)samples. An overview of all (bio)materials collected during the study is shown in Table 1. Furthermore, questionnaires will be sent digitally by email at standardized time points. Table 2 provides a detailed overview of the measures that longitudinally assesses maternal, neonatal or paternal characteristics in the questionnaires.

**Table 1:** Overview of biomaterial samples collected in Lifelines NEXT

Biomaterials	Materials used for collection	Number	Volume (mL)	Sample type^a^	Pregnancy (weeks)		Birth	First year of life (months)							
					12	28		1	2	3	6	9	10	11	12
**Mother**															
Venous blood	EDTA	1	10	Plasma	✕	✕	✕								
	Serum	1	8.5	Serum	✕	✕	✕								
	Serum	1	6	Serum	✕	✕	✕								
	PAXgene	1	2.5	Peripheral blood	✕	✕									
Feces	Cryotube	4	2	Feces	✕	✕	✕	✕	✕	✕	✕	✕			✕
	Swab	1		Feces	✕	✕	✕	✕	✕	✕	✕	✕			✕
Vaginal swab	Swab	1		Vaginal swab			✕								
Placenta (maternal)	RNAlater	2	5	Placenta (maternal)			✕								
Breast milk	Cryotube	15	2	Breast milk				✕	✕	✕	✕	✕			✕
	Swab	1		Breast milk				✕	✕	✕	✕	✕			✕
**Child**															
Umbilical cord blood	EDTA	1	10	Plasma			✕								
	Serum	1	8.5	Serum			✕								
	Serum	1	6	Serum			✕								
	PAXgene	1	2.5	Peripheral blood			✕								
Capillary blood	Blood spot	2		Dry Blood Sample						✕					
	EDTA	4	0.5	Plasma						✕					
				Buffy coat						✕					
Venous blood	EDTA	2	0.5	Plasma											✕
				Buffy coat						✕					✕
	Serum	2	0.5	Serum											✕
	Heparine	2	0.6	Plasma											✕
Placenta (fetal)	RNAlater	2	5	Placenta (fetal)			✕								
Feces	Cryotube	5	2	Feces			✕	✕	✕	✕	✕	✕			✕
	Swab	1		Feces			✕	✕	✕	✕	✕	✕			✕
Nose	Swab RNA	1		Nose swab						✕					✕
	Swab DNA	1		Nose swab						✕					✕
	Swab microbiome	1		Nose swab						✕					✕
Mouth	Swab microbiome	1		Mouth swab						✕					✕
Urine	Peespot	1	1.2	Urine						✕					✕
**Environmental**															
Dust collector	Sheet	2		Dust		✕				✕					
**Father**^b^															
Venous blood	EDTA	1	10	Plasma	✕										
	Serum	1	8.5	Serum	✕										
	Serum	1	6	Serum	✕										
	Heparine	1	9	Plasma	✕										
	PAXgene	1	2.5	Peripheral blood	✕										

^a^All materials stored at − 80 °C, except for the Dry Blood Sample collected at 4 months (room temperature)

^b^Only for non-Lifelines participants, collected at the moment of inclusion

**Table 2:** Overview of measurement in questionnaires

Measurements in questionnaires	Validated instruments used	Pregnancy (weeks)				Birth	First year of life (months)									
		12	18–20	28	32		1	2	3	4	5	6	9	10	11	12
**Mother**																
Work stress *influence on home*-*situation and* vice versa	SWING [24]	✕												✕		
Food preferences *preference or aversion towards foods, physical activity and experiences, avoiding foods*		✕			✕											
Reproductive health—pre-pregnancy *menstrual cycle, birth control, reproductive organs, STDs, fertility, pregnancy history, environmental factors (e.g. smoking), oral health*			✕													
Socioeconomic status *work, education, salary*			✕											✕		
Social adherence *family, friends, colleagues, neighbors and level of satisfaction*	MSPSS, SSL [25, 26]			✕							✕					
Mood/depression *depression, disinterestedness and its effects on functioning*	MINI [27]			✕							✕					
Relation mother/partner *fights and satisfaction with relationship*				✕											✕	
Care tasks *division between partners*				✕							✕					
Bonding mother-to-infant *thoughts and feelings*	MAAS, MPAS [28, 29]			✕							✕					
Medication *dosage, duration, over the counter and prescribed*					✕		✕							✕		
Housing condition—dust *house, ventilation, pets*					✕					✕						
Life events *difficulties and stress associated to life events*	LDI, LTE [30, 31]						✕							✕		
Reproductive health during pregnancy and birth *health, pregnancy disorders, environmental factors(e.g. smoking), birth (including place of birth, interventions and mother’s experiences)*	CEQ [32]						✕									
Food intake	FFQ [33]							✕								
Reproductive health—postpartum recovery									✕							
Parenting style *handling the child and family life situations*										✕						
**Child**																
Crying *Intensity, duration, week average*							✕	✕	✕			✕	✕			✕
Gastrointestinal symptoms *regurgitation, throwing up, defecation pattern and gut complaints*	Rome III [34]						✕	✕	✕			✕	✕			✕
Food intake	FFQ [33]						✕	✕	✕			✕	✕			✕
Medication *dosage, duration, over the counter and prescribed*							✕	✕	✕			✕	✕			✕
Child health *health, house, smoking, pets*										✕						✕
Eczema^a^	SCORAD [20]									✕						✕
Social condition home^a^	HOME [35]									✕						
Lung function^a^	WHISTLER [21, 22]									✕						
Child care *parent, paid babysitter, grandparents*												✕				
Child development *communication, gross and fine motor skills, problem solving, social*	ASQ [36]											✕				✕
Child development *child’s behavior*	IBQ [37]												✕			
Eating behavior															✕	
Social and emotional skills *feelings and behaviors*	BITSEA [38]															✕
Growth and vaccination																✕
**Father**^b^																
Demographics, work, family		✕														
Health *presence of diseases and disorders*	SCL-90 SOM [39]	✕														
Physical activity	SQUASH [40]	✕														
Medication *dosage, duration, over the counter and prescribed*		✕														
Life events *difficulties and stress associated to life events*	LDI, LTE [30, 31]	✕														
Diet	FFQ [41]	✕	✕													
Gastrointestinal symptoms *regurgitation, defecation pattern and gut complaints*	Rome III [34]			✕												
Respiratory health	ECRHS [42]			✕												
Allergies, intoxications				✕												
Visual function	NEI VFQ-25 [43]			✕												
Pain	WPI [44]			✕												
Fatigue	CIS [45]			✕												

^a^Assessed by research nurse

^b^Only for non-Lifelines participants. Items according to the baseline questionnaires from Lifelines, divided in three questionnaires with an interval of 10 weeks, starting at moment of inclusion of the father

Feces and breastmilk are sampled and aliquoted by the participant and stored, along with urine samples, in a freezer in the participant’s home (− 20 °C) until they are collected by the research nurse. The nurse ships the frozen samples to the Lifelines laboratory and biobank, where they are processed and then stored at − 80 °C. Venous blood and umbilical cord blood is drawn by allocated professionals, and samples are immediately stored in the refrigerator either at home or at the hospital (EDTA), kept for 30 min at room temperature and then refrigerated (serum), or kept at room temperature (PAXgene collection tubes). Blood samples are processed and divided into aliquots in the laboratory. Other biomaterials surrounding birth are collected and temporarily stored in a refrigerator (placenta biopsies) or freezer (vaginal swabs) located in either the participant’s homes (for homebirths) or at the hospital where the birth took place. These biomaterials are then transferred to the Lifelines laboratory and biobank within 10 h of birth. All materials are stored in barcoded aliquots at − 80 °C for future research, except for the blood spot collected at 4 months, which is kept at room temperature. All data are stored in a secured data storage environment.

### Measurements of the mother

During pregnancy and the first year after childbirth, participating women are asked to complete eleven different questionnaires on their physical health, psychological health, reproductive health, lifestyle-related behavior and nutritional intake, and social and working conditions (Table 2). During the first home visit, a venous blood sample is drawn by the research nurse. Additionally, during the prenatal period and the first year post-partum, maternal biomaterials are sampled and stored by the participants themselves and collected at home visits by the research nurse (Fig. 1).

During childbirth, the maternity care provider will collect a vaginal swab, a fecal sample and blood from the mother. Immediately after birth, umbilical cord blood and placental tissue are collected according to standardized protocols. The placenta is also weighed and photographed. Maternity care providers also provide a detailed birth report.

### Measurements of the child

Children are included in the birth cohort on the day they are born. During the first year of follow-up, the parents complete eight different questionnaires on their child’s health, development and behavior (Table 2). Seven fecal samples are collected over an interval that starts directly after birth and continues up until the child is 1 year of age. During a home visit at age 4 months, the research nurse will sample capillary blood by a puncture of the child’s heel. At age 12 months, venous blood will be sampled at the pediatrics outpatient clinic at the University Medical Center Groningen (UMCG). Additionally, at the 4- and 12-month home visits, a mouth and a nose sample will be collected for microbiome, DNA and RNA profiling (Table 1). The research nurse will also assess the presence of eczema using the cumulative objective scoring index for atopic dermatitis (SCORAD) [20] (Table 2). During a separate home visit at 4 months of age, lung function tests will be performed with the Single Occlusion Technique (WHISTLER) by a dedicated research nurse [21, 22].

### Measurements of the father

If the father consents to participate in Lifelines NEXT and is also a Lifelines participant, no additional (biomaterial) data needs to be collected. However, if the father is not a participant in Lifelines, he will be embedded in the routine data collection of Lifelines [16]. During a home visit, the research nurse will draw blood and perform anthropometric examinations that conform with the Lifelines protocol (Fig. 1, Table 1) [16]. Additionally, throughout the prenatal period, these fathers will complete three different Lifelines baseline questionnaires with an interval of 10 weeks (Table 2) [16].

### Measurements on the family home environment

Environmental data will be collected by measuring air quality in participant’s homes. Airborne dust will be collected with an electrostatic dust fall collector (EDC) [23] placed during a home visit at 28 gestational weeks and at 4-months postpartum (Table 1). The social conditions at home will also be assessed. Additional data will be collected using self-reported questionnaires that include items about the living condition in the house, smoking and the presence of pets (Table 2). Finally, regional data on air pollution will be collected by extracting geocoded data on postal code level.

### The Newborn initiative

Lifelines NEXT participants also have the opportunity to participate in the Newborn initiative, a public–private project that expands the range of phenotypes measured through agreements with companies developing new technologies while looking at local public health issues. In living lab Newborn, prototypes of innovative products that contribute to health can be tested in real-time home situations. Upon additional consent, these data can be used for product development. The Lifelines NEXT data collection will be enriched with data from continuous measurement devices with built-in apps, e.g. the heartbeat of the mother (healthband^®^), air quality (airvibe^®^) and behavior of the child (uGrow Baby Monitor^®^).

### Study organization

Lifelines NEXT is an initiative of the Departments of Genetics, Obstetrics, Laboratory medicine and Pediatrics of the UMCG. The study is embedded in the organizational structure of the Lifelines cohort study and will use its service center, its laboratory for processing of biomaterials and its infrastructure for digital data collection and storage. All collected biomaterials will be stored in labeled tubes in the Lifelines Lifestore, which currently holds more than 5 million biomaterials of Lifelines participants [16].

The project is governed by a steering committee in which the UMCG Departments of Genetics, Pediatrics, Laboratory medicine, Obstetrics, Community and Occupational Health and Midwifery are represented, as well as the Lifelines organization. The chairman of the steering committee leads the project leaders, maintains contact with external sponsors and is accountable to the supervisory board. Moreover, a supervisory board is in charge consisting of the representatives from the three UMCG Departments of Genetics, Laboratory Medicine and Obstetrics and the board of the UMCG Hereditary Metabolic Diseases Fund. Project leaders from the UMCG and Lifelines work closely to manage the study.

Daily management of Lifelines NEXT is carried out by several members of the Lifelines NEXT research team. A Lifelines research assistant is the primary contact person for all the maternity healthcare providers involved who have questions related to (the data collection of) Lifelines NEXT. The research assistant also extensively monitors the Lifelines NEXT study process of each participant and schedules home visits according to the protocol. Outside normal working hours, a member of the birth team, which consists of medical and midwifery students, is available to support data collection at birth. All birth team members have been trained to collect biomaterials according to protocol, and they arrange the transportation of these biomaterials to the biobank within 3 h. A research nurse is allocated to be the primary contact person for each Lifelines NEXT participant and will perform all the home visits during the study. The research nurse distributes biomaterial collection sets, provides collection instructions and installs devices for environmental data collection. By staying in close contact with the participants, the research nurse acts as a confidant and is instrumental in ensuring that questionnaires are completed and biomaterials collected. Moreover, the research nurse gathers the biomaterials that were collected at home and arranges their transport to the Lifelines laboratory and biobank for processing and storage. All Lifelines NEXT data are stored in a secured data storage environment that utilizes MOLGENIS, a modular suite of web databases for integrating genotype, phenotype and other analyses. Each MOLGENIS database has web user interfaces as well as scriptable interfaces to plug-in R, Java and web services [46]. The handling of data complies with the General Data Protection Regulation (GDPR) [47].

### Statistical power considerations and study size

Based on the Lifelines add-on study Lifelines DEEP (n = 1500), in which integrative analyses have been conducted, a sample size of 1500 subjects will be sufficient to generate novel insights into the preconception, prenatal, birth and early life period [19]. In Lifelines DEEP we had enough power, for instance, to show statistically significant associations between specific microbial compositions and blood lipids [48] and to study various factors influencing microbiome composition and function [49]. These analyses were performed systematically on multiple factors and multiple levels, including microbial diversity, inter-individual distance in composition, individual species and pathway level.

Given a birth rate of 1.6 in the Netherlands [50] and the participation of approximately 40,000 women aged 25–40 years in Lifelines, we expect about 2000 pregnancies per year. Based on previous Lifelines add-on studies, we expect a response rate around 60% [19].

### Harmonization and external database linkages

Harmonization and linkage with baseline and future longitudinally collected Lifelines data, including genome-wide genetic data, will be established for all participants in Lifelines NEXT (i.e. the mother, father and child). This will result in data from up to four generations of the women in Lifelines NEXT. Data of the mothers participating in Lifelines NEXT will be linked with the Dutch Perinatal Data Register, Perined. This perinatal registration data includes three separate databases: one for primary midwife-led care, one for secondary obstetric care and one for neonatal care [11]. These three databases are combined into one national perinatal database via a validated linkage method by the Perinatal Registry Office [51]. The data of the Lifelines NEXT children will be linked with the electronic files of the regional youth and family centers. These centers are entrusted with the national preventive follow-up program of children that monitors the health and development of children from birth until 18 years. This system sees over 95% of all children born in the Netherlands through a comprehensive series of appointments [52]. Moreover, Lifelines NEXT data could be merged and harmonized with data collected by other cohort studies like the Netherlands Kinship Panel Study (NKPS) [53], the birth cohort studying the prevention and incidence of asthma and mite allergy (PIAMA) [54], Trails NEXT [55] and NeoLifeS [56].

## Strengths and limitations

Lifelines NEXT will be a rich resource for research. It is a unique birth cohort that can address critical questions regarding the influence of environmental exposures, social factors, stressors and early-life nutrition on early life development. While the Lifeline NEXT cohort consists of relatively healthy people, it is suitable for research related to (chronic) disease susceptibility (rather than rare diseases). It also enables study of the association of variations in microbiome, metabolomics profiles and (epi)genomics in mothers and children with short- and longer-term health outcomes. The dynamic organization of the study allows us to add additional measurements and to ask new questions during the course of the study. For example, we have already added sampling of feces at all time points in glycerol tubes that allow for later culturing of live bacteria, additional sampling of neonatal feces in week 2 and continuous measurements from connected devices to the study. Further add-on initiatives from additional researchers are also welcomed.

The extended hygiene hypothesis and the EPIgenetic Impact of Childbirth (EPIIC) hypothesis both suggest that factors occurring during the intrapartum and early postnatal period may affect the neonatal immune response or lead to different microbial communities and fetal epigenomic remodeling anomalies [58, 59]. Lifelines NEXT will be able to study different microbial communities of children by metagenomic sequencing, obtaining a comprehensive view of the development of microbial ecosystems in the early life period, as well as its relation to immune, respiratory and metabolic development. It will also be possible to study the change of epigenetic profiles in more detail, for instance that of genes related to allergy in the first years of life [60].

Another strength of Lifelines NEXT is that it offers the possibility to study interactions of immunological, microbial and metabolic maturation processes with environmental factors such as mode of delivery, maternal and early neonatal feeding patterns, type of nutrition (such as breastmilk or formula), indoor and outdoor environmental factors (e.g. exposure to cigarette smoke, air pollution and allergens), use of medication and infections [12, 13, 15, 61–66].

Finally, little attention has been paid thus far to the viral composition of the microbiome: the virome. Because majority of gut viruses are viruses of bacteria (bacteriophages), they are therefore expected to be a major factor in shaping the human microbiome, and could exert an effect on human physiology [67]. So far, little research has been done looking at the role of bacteriophages in the development of gut ecosystem, and their relation to babies health. Through comprehensive analysis, this relatively new area of the role of the virome can be studied in depth.

However, this study had some limitations. At first there is only a limited amount of data available on fathers that were not in the Lifelines study upon the pregnancy of their partners. To meet the need for background information, several baseline questionnaires of the initial Lifelines cohort study are included and will be completed by those new participants. A challenge is to include a representative sample for the Dutch population. As we know, the Lifelines cohort study had, adjusted for differences in demographic composition, a smaller proportion of low educated participants and smokers. However the population was concluded to be broadly representative for the adult population of the north of the Netherlands [57]. Aiming to maintain generalizability we used similar recruitment techniques within Lifelines NEXT.

### Collaboration

We expect inclusion to be completed in 2021, therefore from 2023 onwards biomaterials and data collected in Lifelines NEXT will be available to other researchers. In the meanwhile the Lifelines NEXT consortium welcomes collaboration with other birth cohorts. For example, Lifelines NEXT data could be harmonized and merged with other European and Canadian birth cohorts as proposed in the EUCAN-Connect project [68]. We also welcome add-on initiatives. The Lifelines NEXT cohort study has an open protocol. Interested researchers can submit an application for an additional study or additional (biomaterial) data collection to the steering committee of Lifelines NEXT for approval. Further information can be requested by e-mail: (lifelinesnext@umcg.nl). The Lifelines website (www.Lifelines.nl) provides information about the application process and the data collection, and gives an overview of all available data within Lifelines and publications with Lifelines data.
